# Identification of dental implant systems from low-quality and distorted dental radiographs using AI trained on a large multi-center dataset

**DOI:** 10.1038/s41598-024-63422-z

**Published:** 2024-06-01

**Authors:** Jae-Hong Lee, Young-Taek Kim, Jong-Bin Lee

**Affiliations:** 1 Department of Periodontology, Jeonbuk National University College of Dentistry, 567 Baekje-daero, Deokjin-gu, Jeonju, 54896 Korea; 2 Research Institute of Clinical Medicine of Jeonbuk National University-Biomedical Research Institute of Jeonbuk National University Hospital, Jeonju, Korea; 3 Department of Periodontology, Ilsan Hospital, National Health Insurance Service, Goyang, Korea; 4 Department of Periodontology, Gangneung-Wonju National University College of Dentistry, Gangneung, Korea

**Keywords:** Artificial intelligence, Dental implants, Diagnostic imaging, Deep learning, Oral diseases, Dentistry

## Abstract

Most artificial intelligence (AI) studies have attempted to identify dental implant systems (DISs) while excluding low-quality and distorted dental radiographs, limiting their actual clinical use. This study aimed to evaluate the effectiveness of an AI model, trained on a large and multi-center dataset, in identifying different types of DIS in low-quality and distorted dental radiographs. Based on the fine-tuned pre-trained ResNet-50 algorithm, 156,965 panoramic and periapical radiological images were used as training and validation datasets, and 530 low-quality and distorted images of four types (including those not perpendicular to the axis of the fixture, radiation overexposure, cut off the apex of the fixture, and containing foreign bodies) were used as test datasets. Moreover, the accuracy performance of low-quality and distorted DIS classification was compared using AI and five periodontists. Based on a test dataset, the performance evaluation of the AI model achieved accuracy, precision, recall, and F1 score metrics of 95.05%, 95.91%, 92.49%, and 94.17%, respectively. However, five periodontists performed the classification of nine types of DISs based on four different types of low-quality and distorted radiographs, achieving a mean overall accuracy of 37.2 ± 29.0%. Within the limitations of this study, AI demonstrated superior accuracy in identifying DIS from low-quality or distorted radiographs, outperforming dental professionals in classification tasks. However, for actual clinical application of AI, extensive standardization research on low-quality and distorted radiographic images is essential.

The dental implant system (DIS) has been effectively utilized for several decades in both partially and fully edentulous patients, solidifying its position as a standard treatment modality in the dental field^1^. However, Given the inherent exposure of DISs to mechanical, physiological, and microbiological stimuli within the oral environment, the occurrence of mechanical and biological complications becomes inherently probable^2–4^. Recent systematic reviews have shown that the prevalence of biological complications varies from 1.1 to 85.0%, whereas mechanical complications have a cumulative prevalence of 5.6–7.7%^5,6^.

Mechanical and biological complications associated with DIS require interventions, ranging from repair and component replacement to complete removal from the jaw in severe cases. Before addressing these complications, it is essential to have a comprehensive record of clinical and radiological data on DIS, which can be readily obtained from the patient's dental records^7^. However, when historical dental records are inaccessible due to factors such as practice closures or relocations, the identification of DIS often depends on information derived from dental radiographs.

Prior to the 2010s, the classification of DIS relied primarily on parallel periapical or panoramic radiographs. While widely used, these traditional radiographic methods were limited by their reliance on human visual perception and expertise, which may not adequately differentiate between the various types of DIS that are now available^8,9^. The integration of artificial intelligence (AI) into dental implantology improves diagnostic accuracy and consistency, provides a seamless transition from traditional methods, and overcomes the limitations of human perception to revolutionize DIS classification^10,11^.

Active research is currently underway to identify DISs using AI-driven visual recognition methods, particularly those based on deep convolutional neural network algorithms. Two recent systematic reviews have shown that deep learning algorithms have impressive accuracy rates of 92.2% (95% confidence interval [CI] 90.8–93.5%) and 92.6% (95% CI 90.5–94.6%) in identifying different types of DIS using dental radiographs^10,11^. Furthermore, several studies have shown that the classification accuracy of AI exceeds that of both specialized and non-specialized human experts in dental implantology, and the classification speed provided by AI is up to 19 times faster, reinforcing the potential for clinical application in DIS identification^12,13^.

For the effective application of AI-driven visual recognition, high-quality images in large numbers are essential for training deep learning algorithms. Therefore, research based on dental radiographs has also made significant efforts to obtain validated training datasets, and in particular, several studies have already performed deep learning evaluations based on large datasets consisting of hundreds of thousands of DIS radiographs^13,14^. However, an inherent limitation exists that most studies based on verified DIS datasets may not be truly representative of DIS identification based on low-quality and distorted dental radiographs, which are commonly encountered in actual clinical practice. This research pioneers the evaluation of a state-of-the-art AI model, uniquely trained on an extensive multi-center dataset, to accurately discriminate between different types of DIS in low-quality and distorted dental radiographs. This model will set new standards in diagnostic accuracy and reliability under challenging imaging conditions. We hypothesize that this AI-driven approach will significantly outperform traditional methods, providing a transformative tool for dental diagnostics and potentially improving patient outcomes.

## Materials and methods

### Ethics statement

All procedures were in accordance with the ethics committee and with the principles of the Declaration of Helsinki. This study was approved by the Institutional Review Board of Wonkwang University Daejeon Dental Hospital (approval No. W2104/003-001), and the need for informed consent was waived due to the retrospective nature of the study. This study was conducted in accordance with the Strengthening the Reporting of Observational Studies in Epidemiology (STROBE) and AI in dental research guidelines^15,16^.

### Training and validation datasets

Large-scale and multi-center-based DIS radiographic images, managed and supervised by the National Information Society Agency and the Ministry of Science and Information and Communication Technology, were used to train and validate the deep learning model in this study. The dataset was collected from five college dental hospitals and 10 private dental clinics in 2021 and was released to limited researchers through the AI-HUB platform (www.aihub.or.kr) in 2022. In order to obtain a dataset optimized for deep learning training, each digital imaging and medical communication format-based radiograph was converted to a JPEG file, with a single implant fixture isolated as a single region of interest (ROI) image. Subsequently, each ROI was labeled with the manufacturer, brand and system, diameter and length, placement position, date of surgery, age, and sex using customized image processing, labeling, and annotation tools. The entire dataset was subjected to a rigorous quality assurance process by a board-certified oral and maxillofacial radiologist and a panel of 10 dental experts affiliated with the Korean Academy of Oral and Maxillofacial Implantology to validate brightness, contrast, resolution, image quality, and distortion (see Table S1 of the supplemental information).

The final training and validation datasets consisted of 156,965 panoramic and periapical radiographs representing 10 manufacturers, including Dentium (*n* = 41,096, 26.2%), Dentsply (*n* = 15,296, 9.7%), Dioimplant (*n* = 1530, 1.0%), Megagen (*n* = 7,801, 5.0%), Neobiotech (*n* = 21,260, 13.5%), Nobel Biocare (*n* = 3644, 2.3%), Osstem (*n* = 42,920, 27.3%), Shinhung (*n* = 3376, 2.2%), Straumann (*n* = 4977, 3.2%), and Warantec (*n* = 15,065, 9.6%) (Table 1). The dataset was randomly and equally partitioned into training (*n* = 125,572, 80%) and validation (*n* = 31,393, 20%) subsets. While the total number of training datasets is large, the number of DISs in each is uneven and highly imbalanced. Therefore, before applying the test dataset to the deep learning model, the training subset was augmented tenfold and was used as follows to reduce deviation: random rotations (with a range of 180°), hue adjustments (from  − 0.2 to 0.2), brightness modulation (from  − 0.12 to 0.12), contrast changes (from 0.5–1.5), zoom changes (from 0.5 to 1.5), noise addition (0.05), and horizontal and vertical flips.

**Table 1:** Training and validation datasets consisting of 116,756 panoramic and 40,209 periapical images, comprising 10 manufacturers and 27 types of dental implant systems.

Manufactures	System	Panoramic images (*n* = 116,756)		Periapical images (*n* = 40,209)		Total images (*n* = 156,965)	
Dentium	Implantium	14,993	12.84%	4162	10.35%	19,155	12.20%
	Superline	16,734	14.33%	5207	12.95%	21,941	13.98%
Dentsply	Astra OsseoSpeed TX	13,404	11.48%	571	1.42%	13,975	8.90%
	Xive	667	0.57%	654	1.63%	1321	0.84%
Dioimplant	UF	525	0.45%	273	0.68%	798	0.51%
	UF II	447	0.38%	285	0.71%	732	0.47%
Megagen	Any ridge	217	0.19%	135	0.34%	352	0.22%
	Anyone internal	1640	1.40%	2167	5.39%	3807	2.43%
	Anyone external	1290	1.10%	1263	3.14%	2553	1.63%
	Exfeel external	974	0.83%	115	0.29%	1089	0.69%
Neobiotech	IS I	6708	5.75%	1139	2.83%	7847	5.00%
	IS II	2774	2.38%	104	0.26%	2878	1.83%
	IS III	7594	6.50%	533	1.33%	8127	5.18%
	EB	1890	1.62%	518	1.29%	2408	1.53%
Nobel Biocare	Brånemark	3302	2.83%	342	0.85%	3644	2.32%
Osstem	GS II	1401	1.20%	327	0.81%	1728	1.10%
	SS II	717	0.61%	116	0.29%	833	0.53%
	TS III	19,222	16.46%	10,536	26.20%	29,758	18.96%
	US II	7295	6.25%	2363	5.88%	9658	6.15%
	US III	758	0.65%	185	0.46%	943	0.60%
Shinhung	Luna S	2935	2.51%	441	1.10%	3376	2.15%
Straumann	Standard	1151	0.99%	176	0.44%	1327	0.85%
	Standard plus	741	0.63%	301	0.75%	1042	0.66%
	BL/BLT	1347	1.15%	1261	3.14%	2608	1.66%
Warantec	Hexplant	4568	3.91%	4271	10.62%	8839	5.63%
	Internal	3224	2.76%	2556	6.36%	5780	3.68%
	IT	238	0.20%	208	0.52%	446	0.28%

### Test dataset

The test dataset, which is completely independent of the training and validation datasets, was obtained based on the DIS dataset used in our previous multi-center study^17^. The panoramic and periapical radiographic images were collected from three dental hospitals: Daejeon Dental Hospital, Wonkwang University; Ilsan Hospital, National Health Insurance Service; and Mokdong Hospital, Ewha Womans University. Of the DIS images included in the raw test dataset, low-quality and distorted radiographs were included in the final test dataset for this study according to the following criteria: (1) lack of perpendicular alignment to the implant fixture axis, (2) radiation overexposure, (3) cut off the apex of the implant fixture, and (4) presence of foreign bodies. Consequently, the test dataset used in this study included 586 panoramic and periapical radiographs representing nine different types of DIS, including Dentsply Astra OsseoSpeed TX (*n* = 14, 2.4%), Nobel Biocare Brånemark System MkIII TiUnite (*n* = 12, 2.0%), Dentium Implantium (*n* = 116, 19.8%), Shinhung Luns S (*n* = 8, 1.4%), Straumann SLAactive BL (*n* = 74, 12.6%), Straumann SLAactive BLT (*n* = 20, 3.4%), Straumann Standard Plus (*n* = 8, 1.4%), Dentium Superline (*n* = 90, 15.4%), and Osstem TSIII (*n* = 244, 41.6%) (see Table S2 of the supplemental information).

### DL algorithm

**Figure 1:**
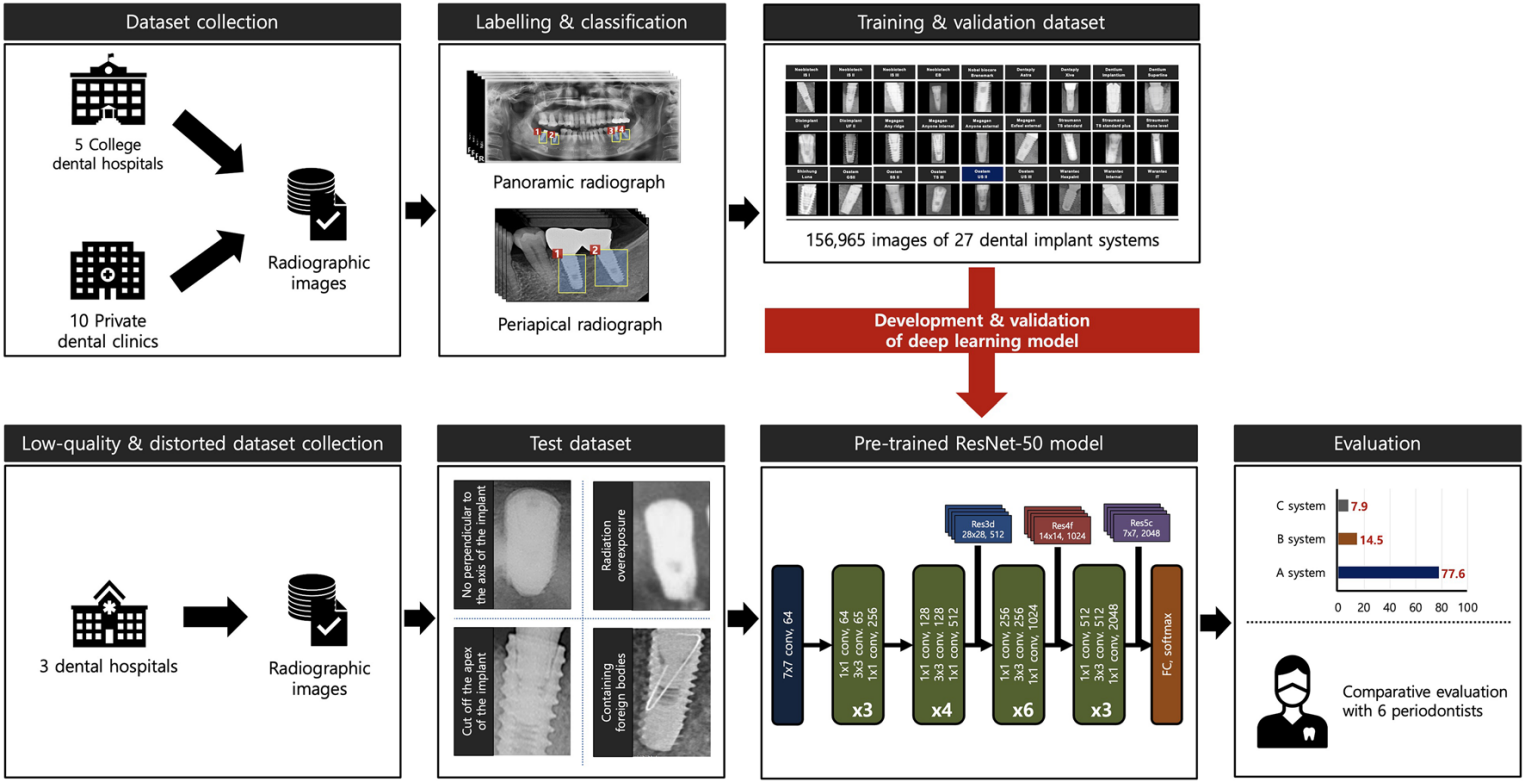
Schematic illustration of the collection, verification, and evaluation of datasets.

ResNet-50 algorithm belongs to the ResNet family of deep convolutional neural network architectures, introduced by He et al. in 2015^18^. The ResNet model has achieved state-of-the-art results in various computer vision benchmarks and has demonstrated strong performance in previous DIS identification studies using two-dimensional dental radiographs^13,19^. Specifically, the ResNet-50 algorithm consisted of 50 deep layers (including one initial convolutional layer, 16 residual blocks of 3 layers each, and one fully connected layer), and these connections bypassed one or more layers and feed the output from one layer directly into a later layer, effectively allowing the model to learn identity functions. All included images were resized to 224 × 224 pixels and entered into a fine-tuned pre-trained ResNet-50 algorithm. All deep learning processes were performed in MATLAB 2023a (MathWorks, Natick, MA, USA) and Python 3.11 (Python Software Foundation, Wilmington, DE, USA) using the TensorFlow framework. The hyperparameter optimization process was carried out systematically using iterative trial and error strategies. Specifically, the Adam optimizer was chosen for its adaptive learning rate capabilities. The batch size was set to 32 to balance computational efficiency and training stability. The learning rate was set to 0.001 to ensure gradual convergence without overshooting the minimum of the loss function. Training was performed for a maximum of 100 epochs. To avoid overfitting and to optimize computational resources, we included an early stopping mechanism that terminated the training process if there was no improvement in the validation set loss for more than 10 epochs (Fig. 1).

### Self-reported questionnaire

In this study, a comparative analysis of the accuracy performance of DIS classification was conducted between AI and five periodontists from Jeonbuk National University Dental Hospital. Based on 586 low-quality and distorted radiographs, a self-report questionnaire was developed by one researcher who was not involved in the study. Pre-classification training and calibration sessions were conducted to improve diagnostic accuracy by aligning periodontists' judgement with established standards. Subsequently, standardized radiographs and illustrations of nine types of DISs were provided prior to the survey, and the survey was completed individually and independently without further information.

### Statistical analysis

Categorical and continuous variables were expressed as frequencies (*n*), percentages (%), 95% CIs, range (minimum–maximum), and median values. Regarding the accuracy assessment of the low-quality and distorted radiographs, the following metrics were used: Accuracy was calculated as (true positive [TP] + true negative [TN])/(TP + TN + false positive [FP] + false negative [FN]); precision was defined as TP/(TP + FP); recall was calculated as TP/(TP + FN); and the F1 score was determined by 2 × (precision × recall)/(precision + recall). Furthermore, the areas under the receiver operating characteristic (ROC) curves (AUCs) and the normalized confusion matrix were presented. All deep learning processes were performed in MATLAB 2023a (Deep Network Designer package, MathWorks, Natick, MA, USA) and Python 3.11 (Keras framework in Python, Python Software Foundation, Wilmington, DE, USA) using the TensorFlow framework.

## Results

**Figure 2:**
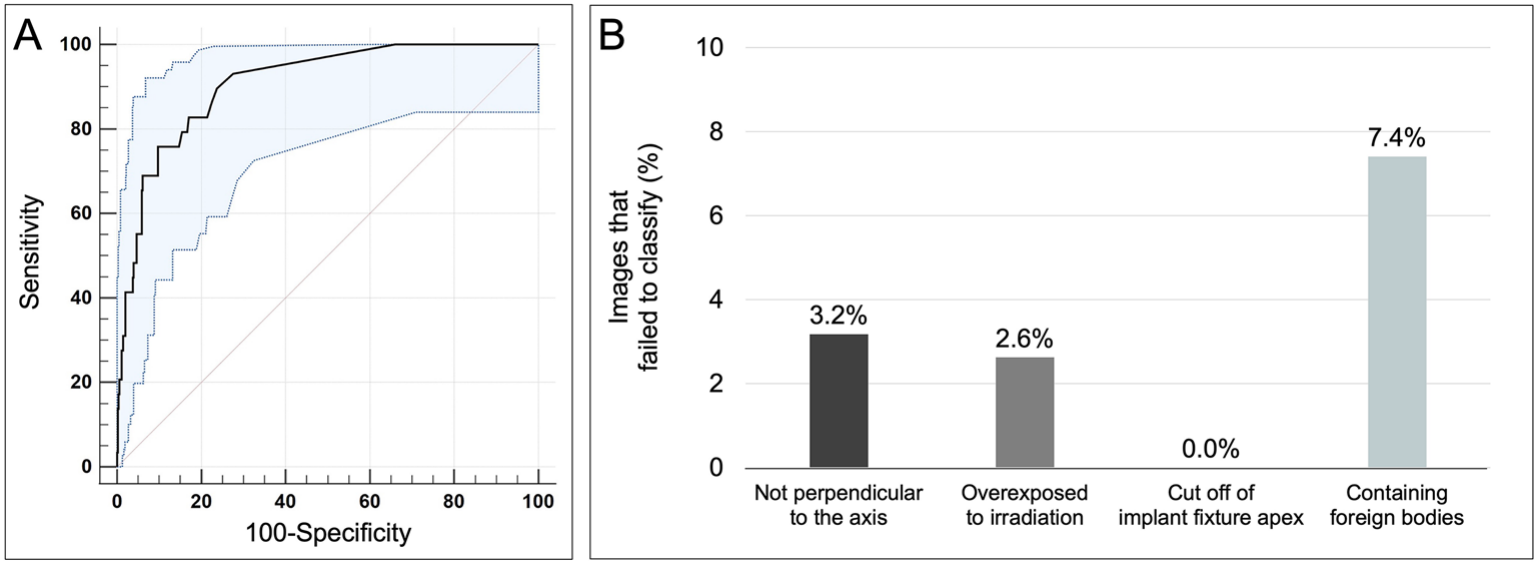
Performance of the deep learning model on 586 low-quality and distorted radiological images. (**A**) Receiver operating characteristic curve and 95% confidence interval for the accuracy of nine different types of dental implant system (DIS) identification, (**B**) Failure to classify DIS based on four categorized low-quality and distorted radiographs.

**Figure 3:**
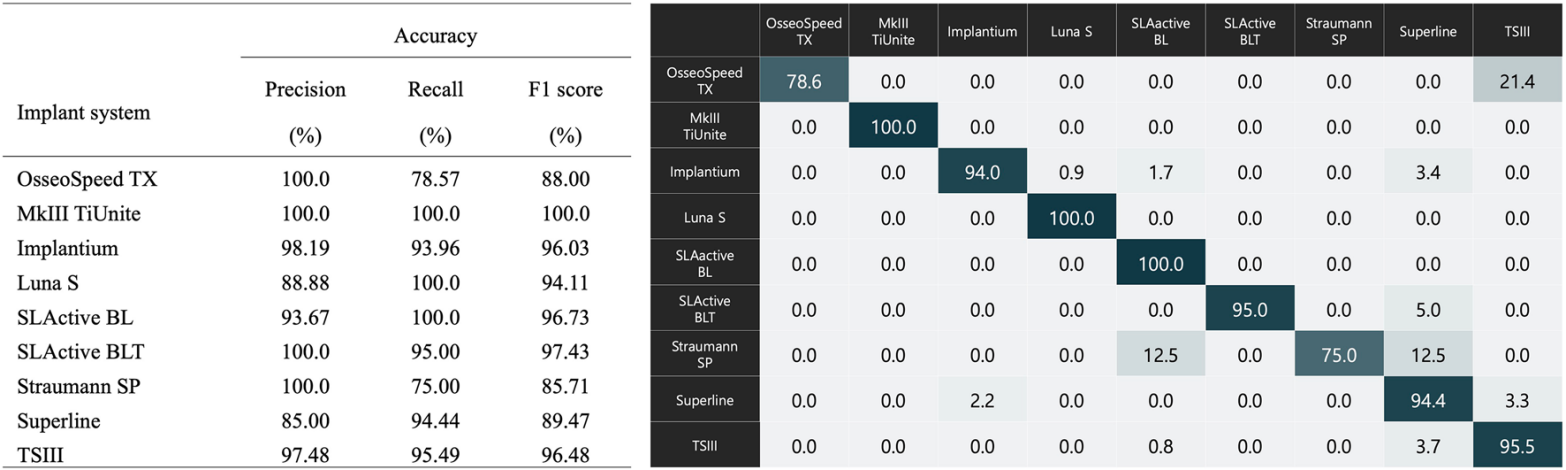
Detailed accuracy performance of each DIS, and normalized multi-label confusion matrix.

**Figure 4:**
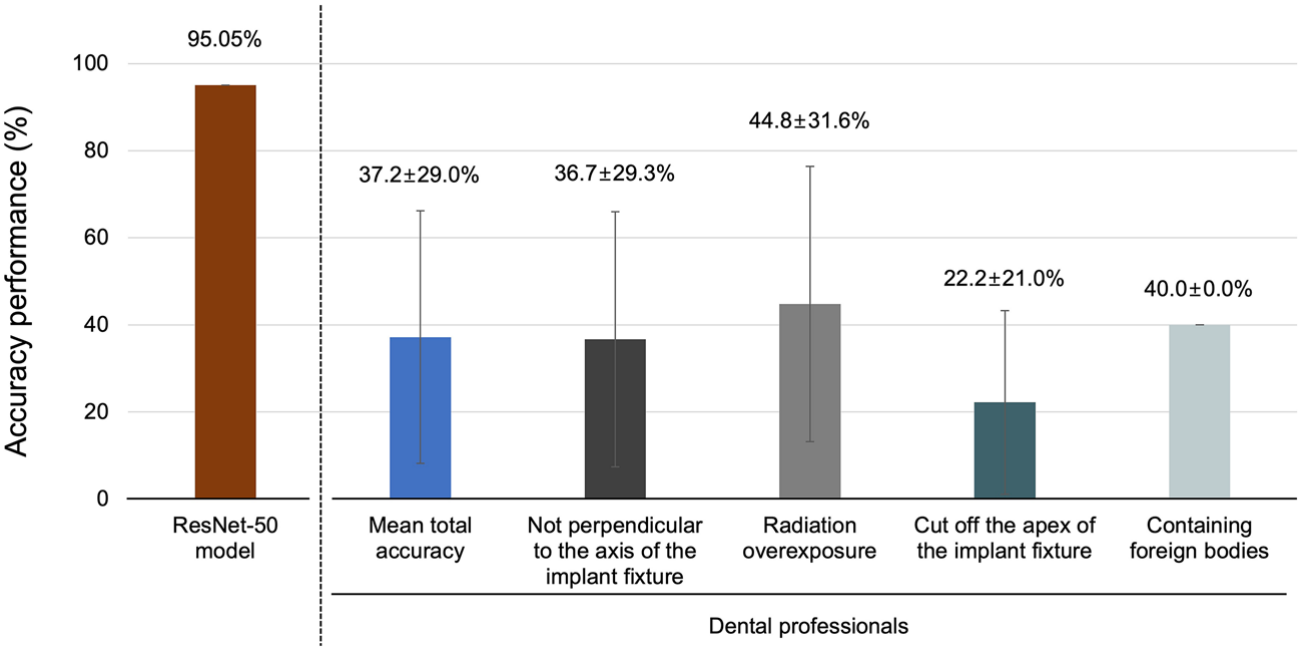
Mean accuracy performance of dental professionals using low-quality and distorted radiography.

The performance evaluation of the ResNet-50 algorithm, based on a test dataset, yielded accuracy, precision, recall, and F1 score metrics of 95.1%, 95.9%, 92.5%, and 94.2%, respectively (Fig. 2). The dataset consisted of 586 distorted radiographic images, categorized as follows: 371 images (63.4%) were not perpendicular to the axis of the implant fixture, 152 (26.0%) showed radiation overexposure, 49 (8.4%) showed a section of the apex of the implant fixture, and 14 (2.4%) contained foreign bodies. The corresponding classification failure rates for these categories were 3.2%, 2.6%, 0.0% and 7.4%, respectively, with no statistically significant difference between the failure rates for each category. When evaluating each of the nine DISs included in the study, the classification accuracy of DL was the highest for MkIII TiUnite (100.0%, precision: 100.0%, recall: 100.0%, and F1 score: 100.0%) and the lowest for Straumann SP (75.0%, precision: 100.0%, recall: 75.0%, and F1 score: 85.7%), with statistically significant differences (*p* < 0.05) (Fig. 3). Based on the training dataset, five periodontists performed the classification of nine different types of DIS, achieving a mean overall accuracy of 37.2 ± 29.0%. The classification accuracy of the dentists was significantly better for cases with radiation overexposure (37.2 ± 29.0%), whereas it was significantly lower for cases in which the apex of the implant fixture was cut off (22.2 ± 21.1%) (Fig. 4).

## Discussion

This study aimed to evaluate the accuracy of AI in identifying DISs based on low-quality and distorted dental radiographs and to compare its accuracy performance with that of human experts. Within the limited research findings, the AI-based deep learning model exhibited a remarkable classification accuracy of 95.05% for identifying nine types of low-quality or distorted DISs, whereas periodontists demonstrated a mean accuracy of 37.2%. The higher accuracy of the AI in this study compared to that in previous studies is likely due to the fact that there were only nine DISs to classify, and the implant systems used in this study were well trained with a high proportion of training and validation datasets^12,13^. AI often outperforms human experts in tasks involving large amounts of ambiguous data or repetitive processing, owing to its ability to work without fatigue and maintain high levels of consistency^20^. These results also suggest that AI can perform efficiently in classifying between different types of low-quality and distorted radiographs in actual clinical practice.

In a previous study based on 801–11,980 radiological images and 3–11 types of DIS, excluding low-quality or distorted images, AI achieved an average classification accuracy of 92.16% (95% CI 90.8–93.5%, min–max 70.75–98.19%), while recent studies using more than 150,000 images, 25 different types of DISs, and the ResNet-50 algorithm have achieved classification accuracies of 82.1–88.5%^10,12–14^. The higher accuracy of the AI in this study compared to previous studies is likely due to the fact that there were only nine DISs to classify, and the implant systems used in this study were well trained with a high proportion of training and validation datasets^12,13^. To the best of our knowledge, most studies have used cropped panoramic and periapical radiographs to train, validate, and test deep learning models for DIS classification, and a significant number of low-quality and distorted images have been excluded in the process of improving the quality of the dataset^21,22^. Therefore, the classification accuracy of AI for different types of low-quality and distorted DIS radiographic images encountered in actual clinical settings has not been properly evaluated to date, which can be considered a significant limitation to employing AI as a decision-aid tool.

Periapical radiography, with its superior clarity and resolution compared to panoramic radiography, is expected to demonstrate statistically significantly higher accuracy when AI is employed for dental radiography^23^. However, previous studies have suggested that the variation in AI classification efficiency between the two imaging modalities falls within the range of 2.3–5.9%, making it challenging to definitively establish the statistical accuracy performance difference^24,25^. Besides these prior observations, the test dataset in this study had a limited number of images. Therefore, to reduce selection bias in the results, we did not evaluate panoramic and periapical radiographs separately.

Previous studies have shown AI's superior accuracy in classifying different types of DISs from dental radiographs compared to that by dentists, specialized and not specialized in implantology^12,13^. One study demonstrated AI's accuracy as 82.0% in classifying 25 types of DISs, significantly outperforming dentists' accuracy rate of 23.5%; another study also reported that AI showed an accuracy of 80.6%, whereas dental professionals had an accuracy of 63.1% in classifying six types of DISs^12,13^. Our results were fairly consistent with those of these past studies. Upon analyzing the detailed classification results of the AI, cases with the apex of the implant fixture cutoff were classified with 100% accuracy, whereas cases involving foreign bodies had the lowest accuracy, achieving a success rate of 92.6%. In contrast, dental professionals showed the lowest classification accuracy (22.2%) when faced with incomplete information about the morphology and shape of DIS owing to the apex of the implant fixture being the cutoff. These results suggest that AI may struggle to identify DIS complications in distorted images caused by the presence of foreign bodies. Therefore, when using AI-based decision support tools in clinical practice, it is important to consider that the classification accuracy of AI may be significantly reduced in the presence of foreign bodies.

Deep learning algorithms are considered to have difficulty classifying images with foreign objects because they lack diverse training data and are unable to detect unexpected distortions. Foreign bodies introduce complexity and diverse features that challenge model accuracy, where balancing sensitivity and specificity can lead to increased false positives or missed subtle anomalies^26^. Therefore, when using an AI-based decision-aid tool in actual clinical practice, one must consider that the classification accuracy of the AI may be significantly lower than the average accuracy performance when foreign bodies are included.

The Straumann SP DIS was the only one of the nine DISs included in this study with a machined surface collar design, allowing dental professionals to easily identify it from other types of DIS with completely different morphology and shape. However, contrary to common visual recognition mechanisms, when assessing the accuracy performance of each DIS included in the test dataset, the AI had the lowest accuracy (75.0%) on the Straumann SP DIS. This is because the image classification process in deep learning models is a black box and involves different cognitive processes than of humans, such as preprocessing the radiographic image, extracting features using convolutional and pooling layers, flattening the resulting output, forwarding it through fully connected layers, and finally classifying the image with the output layer^27–29^. The low AI recognition accuracy of Straumann SP DIS also highlights the need for diverse datasets and the importance of understanding the differences between human and AI recognition, suggesting that AI models need to be continually updated and targeted for improvement based on comprehensive error analysis. Furthermore, out of 156,965 training and validation datasets, only a relatively small number of Straumann SP DIS (*n* = 1042, 0.66%) were used to train the AI, which is likely to contribute to the lower classification accuracy of Straumann SP DIS.

The difficulty that AI systems have in classifying images with foreign objects is often due to a lack of diverse training data that includes such scenarios, leading to challenges in detecting unexpected distortions. The complexity and variety of features presented by foreign objects add further challenges. In addition, the balance between model sensitivity and specificity can affect accuracy, either by increasing false positives or by missing subtle anomalies.

AI can improve clinical decision-making by serving as a proactive diagnostic tool and integrating with electronic dental records to provide real-time insights^30,31^. However, rigorous validation, ongoing performance monitoring, and ethical oversight are essential to ensure the reliability of AI and compliance with dental standards. Future research can improve AI classification accuracy by incorporating more diverse and comprehensive data, refining algorithms with advanced machine learning techniques, and using newer image processing methods such as augmented reality^32^. Hybrid models that combine AI with human expertise can leverage the strengths of both to improve diagnostic accuracy^33,34^. Further development of Explainable AI will help clinicians understand AI decision-making processes, thereby increasing confidence and effectiveness.

This study had a few limitations. First, the number of images used as the test dataset was relatively small compared to the number of images used as training and validation datasets. Future multi-center follow-up studies with more types and images of implant datasets are essential to validate the results of this study. Second, another major limitation of the test dataset was the uneven distribution of DISs and the highly biased representation of the four types of low-quality and distorted images. Third, although the dental radiographs were reviewed twice by a single periodontist to minimize subjective and selective bias, the classification of the four types of low-quality and distorted images remained inherently subjective. Therefore, considerable caution should be exercised in interpreting the results of this study. In addition, the ethical application of AI in future clinical settings will require meticulous accuracy, transparency, rigorous testing, bias mitigation, and strict data security.

## Conclusion

Based on the limited data available, the results of this study highlight the significant potential for AI to improve the accuracy of identifying DIS from challenging dental radiography datasets, particularly when images are of poor quality or distorted. However, this study as conducted under highly specific conditions, which may limit the applicability of AI to general clinical practice. Future research should expand the dataset, ensure a balanced representation of DIS, involve multiple raters, and test the AI under a wider range of clinical conditions to improve its reliability and generalizability.

### Supplementary Information

Supplementary Information.

## Data Availability

The datasets generated and/or analysed during the current study are available in the AI-HUB platform repository, https://aihub.or.kr/aihubdata/data/view.do?currMenu=115&topMenu=100&aihubDataSe=realm&dataSetSn=536.

## Code availability

The code implementing the model is available from the corresponding author on reasonable request.
