# Supplementary material for: Identification of dental implant systems from low-quality and distorted dental radiographs using AI trained on a large multi-center dataset
Source: Sci Rep. 2024 Jun 1;14:12606. doi: 10.1038/s41598-024-63422-z (PMC11144187; doi:10.1038/s41598-024-63422-z)

**< Supplemental information>**

**Identification of dental implant systems from low-quality and distorted dental radiographs through artificial intelligence trained on a large-scale and multi-center dataset**

Jae-Hong Lee^1,*^, Young-Taek Kim^2^, Jong-Bin Lee^3^

^1^Department of Periodontology, College of Dentistry and Institute of Oral Bioscience, Jeonbuk National University, Jeonju, Korea

^2^Department of Periodontology, Ilsan Hospital, National Health Insurance Service, Goyang, Korea

^3^Department of Periodontology, Gangneung-Wonju National University College of Dentistry, Gangneung, Korea

**Table S1.** Detailed information on training and validation datasets

| All included protocols and datasets were supervised and managed by the National Information Society Agency (NIA) under the Ministry of Science and ICT and Korean Academy of Oral and Maxillofacial Implantology (KAOMI). Dental radiographic images were collected from five college dental hospitals and 10 private dental clinics, and the requirement for written informed consent was waived for the use of pre-existing and de-identified dataset. This dataset posted online at the AI-Hub website (https://www.aihub.or.kr) |
| --- |
| **1. Ethics**  The large-scale and multicenter dataset used in current study was approved by the following Institutional Review Board (IRB): Seoul National University Dental Hospital (ERI21024), Yonsei University Dental Hospital (2-2021-0049), Gangnam Severance Dental Hospital (3-2021-0175), Wonkwang University Daejeon Dental Hospital (W2104/003-002), Dankook University Dental Hospital (2021-8-004), and national public IRB (P01-202109-21-020). |
| **2. Consortium composition**  The consortium composition and roles are as follows:   - Host organization (BC&Company Inc.): Project planning, general affairs, coordination of each participating institution, and legal/institutional research. - Participating organization 1 (KAOMI): Dataset collection, and primary verification of the dataset.   - Five college dental hospitals: Seoul National University Dental Hospital (Department of Periodontology), Yonsei University Dental Hospital (Department of Advanced General Dentistry), Yonsei University Gangnam Severance Dental Hospital (Department of Oral and Maxillofacial Surgery), Wonkwang University Daejeon Dental Hospital (Department of Periodontology), and Dankook University Dental Hospital (Department of Periodontology).   - Ten private dental clinics: S-plant Dental hospital, E-Well Dental Clinic, Boston Smart Dental Clinic, Dr. Cho's Dental Implant Clinic, Win Dental Clinic, Yonsei Goun-miso Dental Clinic, Yonsei Haedam Dental Clinic, Seoul Top Dental Hospital, BOA Dental Clinic, and Yonsei Samsung Dental Clinic.   - Dataset verification agency: Not involved in the current study - Participating organization 2 (AiT-Story Corp.): AI solution development and quality control. - Participating organization 3 (Belltechsoft): Data-building program development and quality control. |
| **3. Preprocessing and validation**   - The DICOM format of the periapical and panoramic radiographic images was converted into the de-identified and anonymized JPEG format. - The region of interest (one implant fixture per one image) was cropped and labeled, including the manufacturer name, the brand of the system, diameter, length, and placement position based on electronic medical and dental records. - Radiographic image quality inspections were conducted and validated by a board-certified oral and maxillofacial radiologist who was not involved in the dataset collection procedure. - All procedures, including preprocessing and validation, were performed using custom image processing, labelling, and annotation tools (BC & Company Inc, Seoul, Korea). |
| **4. Policy for data use and restrictions**   - The dataset provided by the AI-Hub was established as part of the “Intelligence Information Industry Infrastructure Creation” project of the Ministry of Science and ICT and the NIA. - All rights including tangible and intangible outcomes, such as datasets, AI application models, sources of data authoring tools, and various manuals, are reserved by the NIA. - Any commercial or non-commercial use of the dataset by organizations or individuals requires additional approval from the NIA. |

**Table S2.** Number of panoramic and periapical radiographs for each dental implant system used in the test dataset

| Manufactures | Classification | Panoramic images  (*n* = 45) | | Periapical images  (*n* = 540) | | Total images  (*n* = 586) | |
| --- | --- | --- | --- | --- | --- | --- | --- |
| Dentsply Astra OsseoSpeed TX | lack of perpendicular alignment to the implant fixture axis | 2 | 4.4% | 9 | 1.7% | 11 | 1.9% |
|  | radiation overexposure | 0 | 0.0% | 3 | 0.6% | 3 | 0.5% |
|  | cut off the apex of the implant fixture | 0 | 0.0% | 0 | 0.0% | 0 | 0.0% |
|  | presence of foreign bodies | 0 | 0.0% | 0 | 0.0% | 0 | 0.0% |
| Nobel Biocare Brånemark System MkIII TiUnite | lack of perpendicular alignment to the implant fixture axis | 2 | 4.4% | 10 | 1.9% | 12 | 2.0% |
|  | radiation overexposure | 0 | 0.0% | 0 | 0.0% | 0 | 0.0% |
|  | cut off the apex of the implant fixture | 0 | 0.0% | 0 | 0.0% | 0 | 0.0% |
|  | presence of foreign bodies | 0 | 0.0% | 0 | 0.0% | 0 | 0.0% |
| Dentium Implantium | lack of perpendicular alignment to the implant fixture axis | 8 | 17.8% | 72 | 13.3% | 81 | 13.8% |
|  | radiation overexposure | 2 | 4.4% | 32 | 5.9% | 34 | 5.8% |
|  | cut off the apex of the implant fixture | 0 | 0.0% | 0 | 0.0% | 0 | 0.0% |
|  | presence of foreign bodies | 0 | 0.0% | 1 | 0.2% | 1 | 0.2% |
| Shinhung Luns S | lack of perpendicular alignment to the implant fixture axis | 0 | 0.0% | 8 | 2.5% | 8 | 1.4% |
|  | radiation overexposure | 0 | 0.0% | 0 | 0.0% | 0 | 0.0% |
|  | cut off the apex of the implant fixture | 0 | 0.0% | 0 | 0.0% | 0 | 0.0% |
|  | presence of foreign bodies | 0 | 0.0% | 0 | 0.0% | 0 | 0.0% |
| Straumann SLAactive BL | lack of perpendicular alignment to the implant fixture axis | 3 | 6.7% | 35 | 6.5% | 38 | 6.5% |
|  | radiation overexposure | 3 | 6.7% | 29 | 5.4% | 32 | 5.5% |
|  | cut off the apex of the implant fixture | 0 | 0.0% | 4 | 0.7% | 4 | 0.7% |
|  | presence of foreign bodies | 0 | 0.0% | 0 | 0.0% | 0 | 0.0% |
| Straumann SLAactive BLT | lack of perpendicular alignment to the implant fixture axis | 0 | 0.0% | 14 | 2.6% | 14 | 2.4% |
|  | radiation overexposure | 1 | 2.2% | 5 | 0.9% | 6 | 1.0% |
|  | cut off the apex of the implant fixture | 0 | 0.0% | 0 | 0.0% | 0 | 0.0% |
|  | presence of foreign bodies | 0 | 0.0% | 0 | 0.0% | 0 | 0.0% |
| Straumann Standard Plus | lack of perpendicular alignment to the implant fixture axis | 0 | 0.0% | 5 | 0.9% | 5 | 0.9% |
|  | radiation overexposure | 1 | 2.2% | 2 | 0.4% | 3 | 0.5% |
|  | cut off the apex of the implant fixture | 0 | 0.0% | 0 | 0.0% | 0 | 0.0% |
|  | presence of foreign bodies | 0 | 0.0% | 0 | 0.0% | 0 | 0.0% |
| Dentium Superline | lack of perpendicular alignment to the implant fixture axis | 5 | 11.1% | 49 | 9.1% | 54 | 9.2% |
|  | radiation overexposure | 2 | 4.4% | 9 | 1.7% | 11 | 1.9% |
|  | cut off the apex of the implant fixture | 1 | 2.2% | 18 | 3.3% | 19 | 3.2% |
|  | presence of foreign bodies | 1 | 2.2% | 5 | 0.9% | 6 | 1.0% |
| Osstem TSIII | lack of perpendicular alignment to the implant fixture axis | 7 | 15.6% | 141 | 26.1% | 148 | 25.3% |
|  | radiation overexposure | 3 | 6.7% | 60 | 11.1% | 63 | 10.8% |
|  | cut off the apex of the implant fixture | 3 | 6.7% | 23 | 4.3% | 26 | 4.4% |
|  | presence of foreign bodies | 1 | 2.2% | 6 | 1.1% | 7 | 1.2% |

**Appendix Figure 2.** Detailed information of accuracy performance of ResNet-50 architecture. In this study, we used pretrained and fine-tuned ResNet-50 deep convolutional neural network architecture as classification model. All input radiographic images of ROI are resized to 224x224 pixels. Data augmentation with random rotations (range of 20°), distortions, zooms (range of 0.5–1.5), and horizontal and vertical flips was applied. ResNet-50 consists of five stages with 50 deep layers. The first stage consists of the convolution layer, batch normalization layer, rectified linear unit activation layer, and maximum pooling layer. The second to fifth stage consists of combined convolution block and identity block. The output layer consists of the average pooling layer, FC layer, and Softmax-based classification. The ResNet-50 model was run on Python 3.8 (Python Software Foundation, Wilmington, DE, USA) using TensorFlow and Keras frameworks. We optimize the hyperparameters as follows using trial and error strategies.: optimizer, Adam; number of training epochs, 100; batch size, 128; learning rate, 0.001.

(a) Comparison of the training, validation, and cross-entropy curves for the pretrained models for classification of dental implant systems. Training and validation ware performed for 100 epochs, with each epoch representing one pass through the entire training and validation dataset.


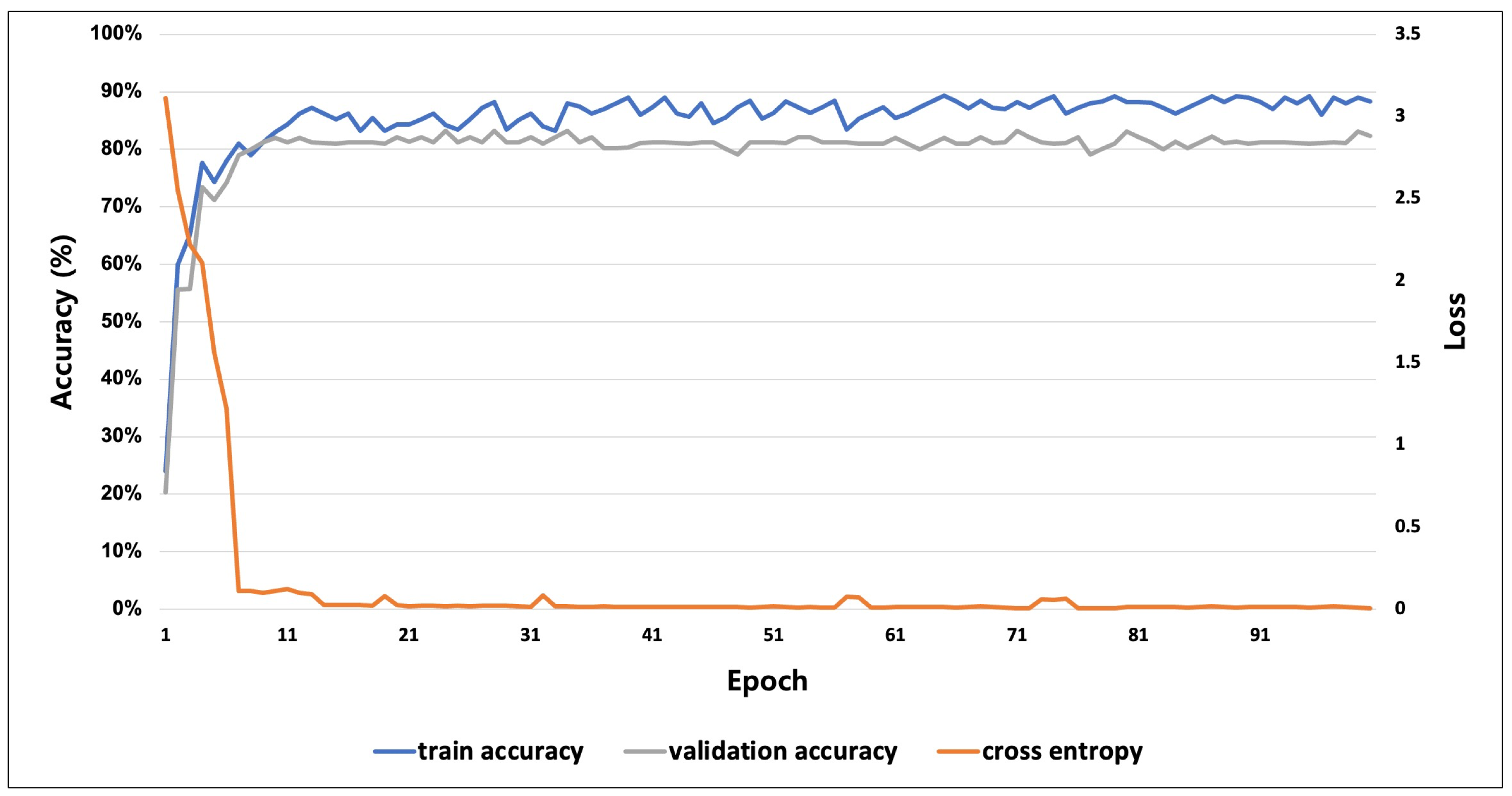

Supplement: Supplementary file 1 — Supplementary Information. [file 41598_2024_63422_MOESM1_ESM.docx]
